# Facile fabrication of Fe-TiO_2_ thin film and its photocatalytic activity

**DOI:** 10.1007/s11356-021-17425-2

**Published:** 2021-11-20

**Authors:** Almudena Aguinaco, Beatriz Amaya, Milagrosa Ramírez-del-Solar

**Affiliations:** 1 Departamento Física de la Materia Condensada and Instituto de Microscopía Electrónica y Materiales (IMEYMAT), Universidad de Cádiz, 11510 Puerto Real, Cádiz Spain; 2 Departamento Ciencias de los Materiales e Ingeniería Metalúrgica y Química Inorgánica and Instituto de Microscopía Electrónica y Materiales (IMEYMAT), Universidad de Cádiz, 11510 Puerto Real, Cádiz Spain

**Keywords:** Nanoparticles, Titanium oxide, Fe-doping, Thin film, Solar photocatalysis

## Abstract

Fe^3+^-TiO_2_ (Fe-TiO_2_) thin films were successfully prepared using a “sandwich” approach. TiO_2_ NPs were doped with different Fe^3+^ content (0.05%, 0.1%, 0.2% molar ratio), and the modified TiO_2_ NPs were deposited on glass flat support by dip coating.

Structural, morphological, optical, and photocatalytic properties of Fe-TiO_2_ thin films were studied. XPS spectra confirm the presence of Ti, Fe, O, and defective –OH groups at the material surface. The Fe 2p spectrum demonstrates the existence of Fe^3+^. SEM images indicate that the incorporation of Fe^3+^ deforms in some degree the homogeneity of the TiO_2_ system. Additionally, incorporation of Fe^3+^ ions to the network creates an impurity band near the VB due to the oxygen vacancies, resulting in the reduction of the effective optical band gap. Photocatalytic activity of fabricated thin films in the elimination of sulfamethoxazole (SMT) follows pseudo first-order kinetics. The highest SMT removal yields were achieved using the sample with 0.05%Fe. Additionally, the use of greater thicknesses improves the removal performance. However, material detachment limits the maximum usable value around 6 µm.

Finally, stability and reusability of catalysts were confirmed studying the photocatalytic activity over three cycles and evaluating that no Fe^3+^ leaching occurred.

**Graphical abstract:**

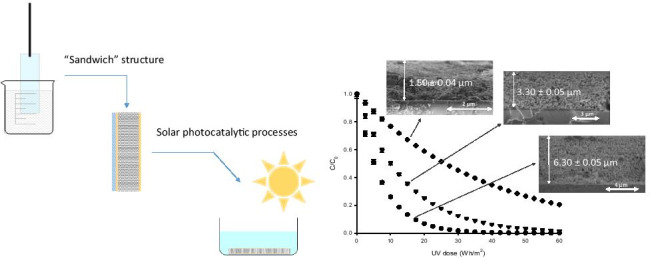

## Introduction

The most widely described photocatalyst in the bibliography is TiO_2_. However, this catalyst presents a band gap value of 3.2 eV, which limits to the UV region the effective radiation wavelength range in order to activate the photocatalyst (Checa et al. 2019). A second disadvantage for its use as catalyst is the high degree of charge carrier recombination characteristic of TiO_2_ (Asahi et al. 2014). Out of all possibilities, coupling TiO_2_ with transition metals such as Fe is a promising way for modifying the photo-absorption properties of TiO_2_ towards the visible range, which is the most intense in the solar spectrum, and at the same time, electron–hole recombination rate is decreased. Iron is an appropriate candidate because Fe ions might easily be incorporated into the TiO_2_ crystal lattice, owing to the fact that radius of Fe^3+^ (0.64 Å) is similar to that of Ti^4+^ (0.68 Å) (Nasralla et al. 2013). Fe_2_O_3_, having a low band gap (∼ 2.4 eV), acts as the sensitizing material in order to absorb the visible light (Wannapop et al. 2021). Moreover, Fe^3+^ is involved in the separation of photo-generated h^+^/e^−^ pairs which is due to the energy level of Fe^3+^/Fe^2+^ which is below the conduction band edge of TiO_2_; the Fe^3+^ can trap the photo-generated electrons. Altogether, Fe^3+^ traps the photogenerated holes (h^+^) due to the energy level of Fe^4+^/Fe^3+^ which is above the valence band edge of titanium dioxide (Khasawneh et al. 2021).

Regarding photocatalyst morphology, we have to consider some drawbacks of using it in his powder form: (1) separation of powder from water is difficult, requiring a filtration stage after the treatment, which significantly increases the cost of the process, and (2) the suspended powder tends to aggregate especially when high concentrations are used (Mahadik et al. 2014). Therefore, to overcome these disadvantages, it is recommended to use the photocatalysts in the form of thin films (it has the additional advantage of saving material since a small quantity is required to complete a sheet of nanometer thickness, and in addition, it is expected that they can be reused during many cycles). However, there are few studies about the use of Fe-TiO_2_ thin films for the elimination of contaminants in water by applying solar photocatalysis processes.

For example, Wannapop et al. (2021) studied the photocatalytic degradation of rhodamine B in aqueous solutions by using Fe_2_O_3_/TiO_2_ films were synthesized by a hydrothermal method. Nevertheless, authors utilized 365-nm LED black light blue instead of solar energy, and they do not study the influence of the thickness in the photocatalytic process.

In this work, we are focused on the optimization of a synthetic pathway for the production of Fe^3+^-TiO_2_ thin films with enhanced solar photocatalytic activity for the removal of pharmaceutical compounds from water.

Among all the chemical categories related to pharmaceuticals, antibiotics are one of the most relevant due to their extensive use and their ubiquitous nature as environmental contaminants. In addition to many other noxious effects, the presence and uncontrolled disposal of antibiotics in the environment may also accelerate the development of antibiotic resistance genes into bacteria, which represents health risks to humans and animals (Aba-Guevara et al. 2017).

Sulfamethoxazole (SMT) is a sulphonamide antibiotic, which is one of the common pollutants in surface water. For that reason, in recent years, researchers have developed a variety of treatment methods, including biological, physical, and chemical oxidation to remove trace sulphonamide antibiotics from water environment (Wang et al. 2020). However, the photodegradation of pharmaceutical compounds (e.g., SMT) using Fe_2_O_3_-TiO_2_ photocatalyst was rarely investigated in the literature (Khasawneh et al. 2021), and no studies using Fe_2_O_3_-TiO_2_ thin film has been developed to our knowledge. Thus, in this work, we attempt to fill the research gap and contribute towards investigating the solar photocatalytic degradation of pharmaceuticals using Fe^3+^-TiO_2_ thin films.

## Materials and methods

### Chemicals

Fe(NO_3_)_3_·9H_2_O used to prepare Fe-TiO_2_ NPs was purchased from Sigma-Aldrich (analytic grade) and used as received. Tetrabutyl orthotitanate (TBOT) C_16_H_36_O_4_Ti (98%) and TiO_2_ nanoparticles (P25, 99.5%) were obtained from Sigma-Aldrich. Acetylacetone C_5_H_8_O_2_ (99.5%) was received from MERK. Ethanol C_2_H_6_O (99.5%) was purchased from Panreac (ITW companies).

To assess possible iron leaching, total iron concentration was determined using the ferrozine reagent purchased from Fisher.

SMT used in the photocatalytic experiments was obtained from Sigma-Aldrich. These experiments were carried out using MilliQ water.

### Preparation of Fe-TiO_2_ thin film photocatalyst

#### Preparation of modified Fe-TiO_2_ nanoparticles

0.3 g of Fe (NO_3_)_3_·9H_2_O was dissolved in 100 mL of MilliQ water under magnetic stirring. Following this, in order to lead to a 0.05, 0.1, or 0.2% Fe/TiO_2_ molar ratio, a volume amount of the prepared solution was added, drop by drop, to 3 g of TiO_2_ NPs, forming a slurry that was finally dried at 110 °C for 24 h. Finally, samples were annealed at 400 °C for 1 h.

#### Preparation of photocatalyst films

**Fig. 1:**
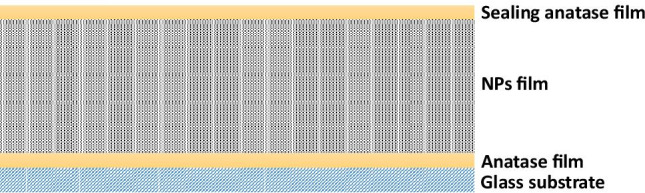
Schematic presentation of TiO_2_ coatings in the proposed “sandwich” structure

For the preparation of the photocatalyst coatings on flat glass supports, we followed a multilayer approach proposed by our group which is described in a previous paper (Rueda-Márquez et al. 2020). According to the methodology, the structure of the coatings responds to a “sandwich” structure where a photocatalyst film is delimited at both sides by anatase films, as shown in Fig. 1.

Conventional microscope glass holders were used as support, and “sandwich” structures were prepared by dip coating technique using a homemade apparatus and a precisely controlled speed of 100 mm/min.

Firstly, TiO_2_ sol precursor was prepared by hydrolysis of tetrabutyl orthotitanate (TBOT) in a solution containing acetylacetone, which is used as a chelating agent to reduce titanium alkoxide functionality and reactivity, and ethanol, as a solvent. Acidified water (HNO_3_, pH = 1) was added drop wise to this solution under stirring, and finally, the solution was diluted with additional ethanol for a final molar ratio TBOT:acetylacetone: H_2_O: EtOH of 1:0.5:2:35. After stirring for 1 h, sol was aged for 100 h at room temperature in a closer container before deposition. Resulting titania sols were stable for several months when were stored in the fridge at 4 °C.

This sol was used to deposit a single TiO_2_ gel thin film on the cleaned glass substrates and dried at 150 °C. As showed in previous papers (Blanco et al. 2018, 2015), this film once annealed will crystallize preferentially as anatase.

Secondly, Fe-TiO_2_ NPs dispersed in acetylacetone-ethanol solution (4:1 vol ratio, 40 g/L) by high-power ultrasonic probe (20 kHz) were deposited on the top of the TiO_2_ gel thin film. The first TiO_2_ gel thin film favors the adherence of the NPs film to the substrate. Thickness of this Fe-TiO_2_ NPs layer was varied by means of the number of depositions performed. To improve the compactness of the layer, samples were dried at 150 °C for 30 min after each layer deposition. Finally, the coating was sealed with another TiO_2_ gel thin film, and samples were annealed at 400 °C for 1 h. The oven was programmed to raise the temperature of 5 °C each min until the desired temperature was achieved. The final annealing leads a high degree of anatase crystallization of the TiO_2_ gel deposited layers. For this reason, in Fig. 1, the first and the last layers in the “sandwich” structure are called anatase film. The anatase top layer plays a dual role as part of the photocatalyst and as a sealing layer for the lower Fe-TiO_2_ layer (Rueda-Márquez et al. 2020). Additionally, TiO_2_ NPs “sandwich” structure films were also prepared using TiO_2_ NPs instead of Fe-TiO_2_ NPs. This sample was used as a reference to determine the advantages of incorporating Fe^3+^ ions when solar photocatalysis processes are applied.

### Photocatalyst characterization

X-ray photoelectron spectroscopy (XPS) measurements were performed using a Kratos Axis Ultra DLD spectrometer, using a monochromatic Al Kα radiation at 1486.6 eV and X-ray power of 150 W. The analyzer was operated in constant analyzer energy transmission (CAE) mode, with pass energy of 20 eV. Surface charging effects were compensated by making use of the Kratos coaxial neutralization system. The binding energy (BE) scale was calibrated with respect to the C 1 s signal at 284.8 eV. XPS data analysis was performed by using CasaXPS Software, version 2.3.19rev1.1 m (Neal Fairley, Casa Software Ltd., UK).

Morphology and thickness of prepared thin films were followed by scanning electron microscopy (SEM), recording cross-section images obtained using the secondary electron detector of a FEI Nova NanoSEM 450 scanning microscope, with field effect electron emission gun, working at a voltage of 20 kV and a working distance of 4.6 mm.

In order to evaluate the influence of the Fe^3+^ incorporation to the titania network on the optical band gap energy, UV–Vis diffuse reflectance measurements were carried out using an Agilent Cary 5000 UV–Vis–NIR double-beam spectrophotometer. The spectra in the 200 to 800 nm range were registered in an integrating sphere. The resulting diffuse reflectance spectra were transformed into apparent absorption spectra using the Kubelka–Munk function (*F(R)*). The indirect optical band gap of the materials was determined through the construction of Tauc plots by plotting (*F*(*R*)*hν*)^*n*^ against (*hν*), with *n* = 1/2. The optical band gap was obtained by extrapolating the linear part of this plot to the energy axis.

### Photocatalytic experiments

**Fig. 2:**
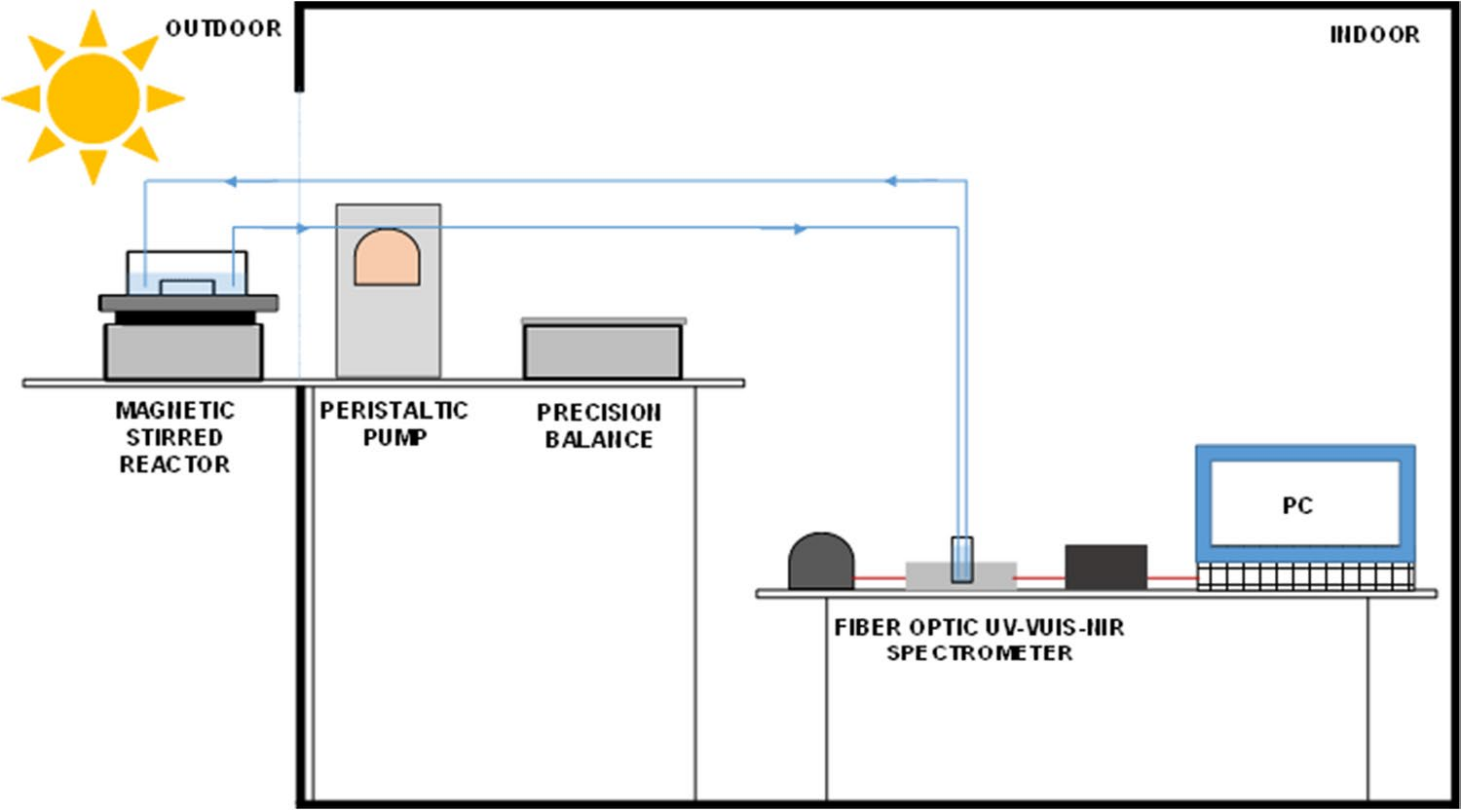
Schematic representation of the experimental set up for photocatalytic experiments

Experiments were carried out using a custom-made photocatalytic activity system, consisting of a magnetic-stirred reactor exposed to sunlight, equipped with a recirculating peristaltic pump (Selecta Percom N-M) and coupled to an Avantes fiber optic UV–Vis-NIR spectrometer (Ocean Optics DT-Mini-2GS light source) that allows continuous monitoring (Fig. 2).

For each experiment, 20 mL of an aqueous solution of SMT was charged into a glass photoreactor (5.6 mm diameter). The initial contaminant concentration was 5 ppm. Also, a Fe-TiO_2_ thin film (2.5 × 2.8 cm) was placed into the reactor. Experiments were conducted at the University of Cadiz, Campus of Puerto Real (Spain, local latitude 36º31′ N) on sunny days. The intensity of solar radiation (UVA and UVB) was measured by employing a radiometer UV34, PCE Iberica, Spain. Thus, solar radiation was evaluated in terms of solar UV irradiance, which is defined as the rate at which solar radiant energy is incident on a surface per unit area of surface (W/m^2^).

Absorbance measurements were determined using a UV–Vis-NIR fiber optic spectrometer, and its evolution over radiation dose was monitored thanks to a peristaltic pump that recirculated (6 mL/min) the contaminant solution treated by solar photocatalysis.

For comparison of solar test results, cumulative UV dose was calculated as shown in Eq. (1). Cumulative UV energy is frequently used for applications in solar reactors (Rueda-Márquez et al. 2020; Sichel et al. 2007):

1 $${Q}_{UV,n}={Q}_{UV, n-1}+\sum \left({UV}_{n}\cdot \Delta t\right)\left({~}^{{V}_{i}}\!\left/ \!\!{~}_{{V}_{T}}\right.\right)$$

where *Q*_UV*n*_, *Q*_UV*n*−1_ is the cumulative UV dose at instant *n* and *n* – 1, respectively; Δ*t*_*n*_ the time interval between two sampling times; UV_*n*_ the average incident radiation for each time interval (W/m^2^); *Vi* the illuminated volume of the reactor; and *V*_*T*_ the total volume of the reactor.

The experimental results can also be expressed in terms of standardized *t*_30W_ solar UV radiation, whereby the actual experimental duration is normalized mathematically (see Eq. (2)) to a hypothetical constant UV radiation intensity of 30 W/m^2^ by introducing correction factors according to radiation intensity (Malato et al. 2002). A UV intensity of 30 W/m^2^ resembles conditions around a sunny noon in the vicinity of Cádiz:

2 $${t}_{30W,n}={t}_{30W, n-1}+\sum \left(\frac{{UV}_{n}}{30}\cdot \Delta t\right)\left({~}^{{V}_{i}}\!\left/ \!\!{~}_{{V}_{T}}\right.\right)$$

where *t*_30W,*n*_ *t*_30W,*n*−1_ is the normalized illumination time of samples at times *n* and *n* – 1.

Thus, the use of cumulative dose or standardized time would allow not only the comparison with other solar photocatalytic experiments but also the possible combination of data from several days’ experiments.

Finally, to evaluate if Fe^3+^ leaching occurred, total iron concentration was determined by the ferrozine method (Stookey 1970) that, briefly, consisted in the reduction to Fe(II) and further formation of a violet complex (ε_565nm_ = 27,044 M^−1^ cm^−1^) with the ferrozine reagent. Absorbance measurements at 565 nm were carried out using an UV–visible spectrophotometer PERKIN ELMER (Lambda 19).

## Results and discussion

### Material characterization

**Fig. 3:**
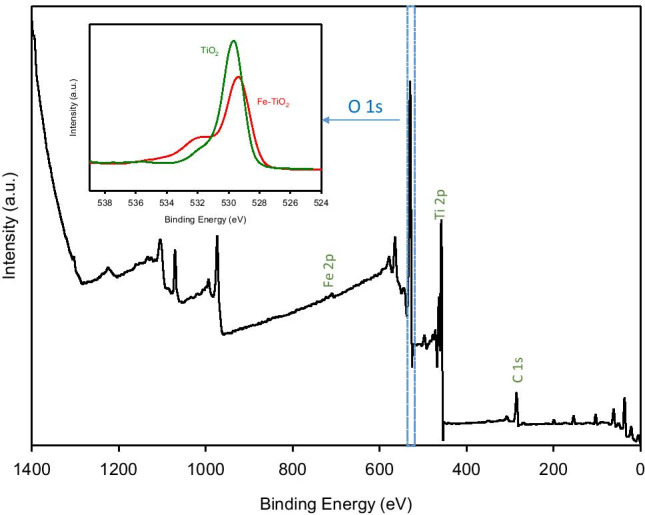
XPS survey scan spectra of 0.2%Fe-TiO_2_. Embedded figure: O1s-scan spectra of 0.2%Fe-TiO_2_ (red line) and TiO_2_ (green line) samples

X-ray photoelectron spectroscopy was performed to investigate the electronic features and elemental composition of the prepared materials. As example, the XPS survey spectrum of 0.2%Fe-TiO_2_ sample is illustrated in Fig. 3, where the main signal peaks corresponding to Ti, Fe, and O are labelled.

**Fig. 4:**
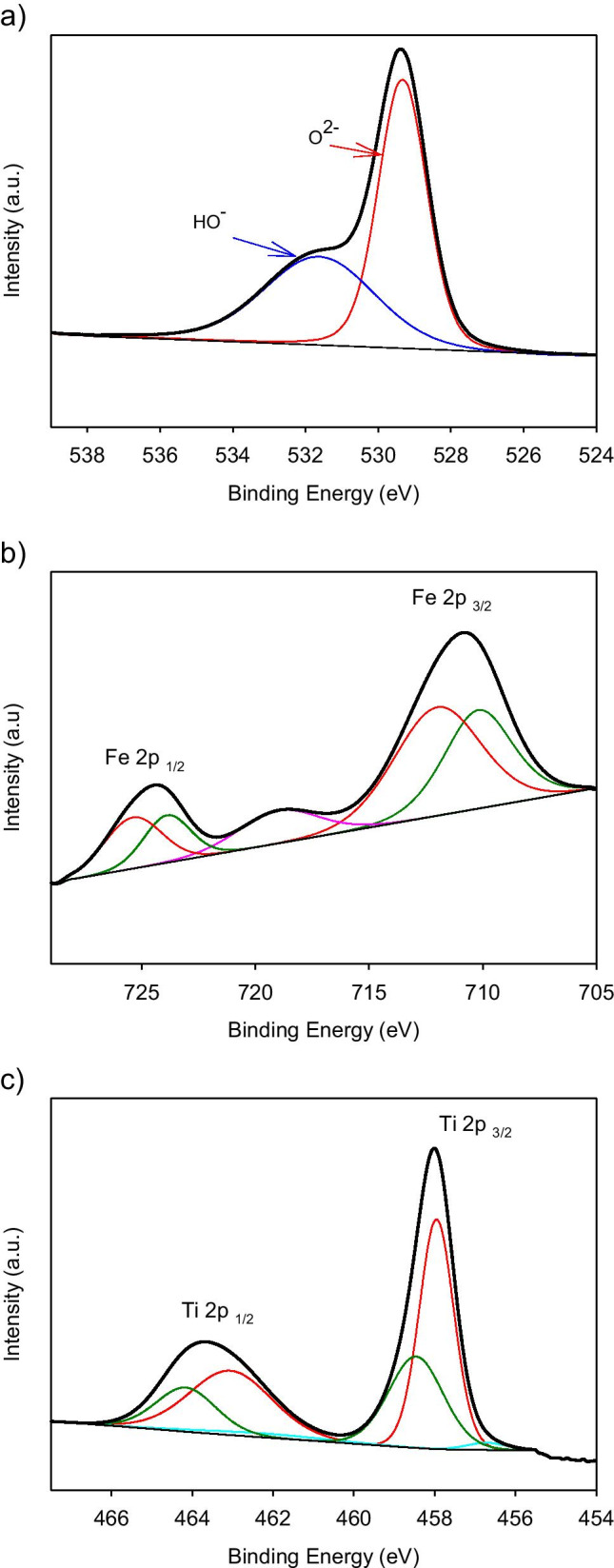
XPS spectra of curve fitting for (**a**) O 1 s, (**b**) Fe 2p and (**c**) Ti 2p

High-resolution XPS signal and their deconvolution are presented in Fig. 4a–c. The surface O 1 s peak was characterized by an asymmetric shape (Fig. 4a), with a marked broadening on the high BE side. According to the literature, the peak at 529.3 eV was ascribed to lattice oxygen in Fe-Ti oxides (Khasawneh et al. 2021; Visentin et al. 2011). On the other hand, the shoulder at 531.7 eV could be associated to oxygen of the defective –OH groups (Palanisamy et al. 2013).

The Fe 2p spectrum (Fig. 4b) demonstrates the existence of Fe^3+^. Specifically, Fe 2p_3/2_ at 712 eV, Fe 2p_1/2_ at 725.4 eV, and the satellite peak present at 719 eV are characteristics for Fe^3+^ ions in Fe_2_O_3_ (Pham et al. 2014; Suresh et al. 2014). The fitted Fe 2p_3/2_ peak at 710.2 eV can be assigned to Fe^3+^ state in the Ti–O-Fe bond, and the absence of the Fe 2p 3/2 peak at about 709.3 eV suggests that no Fe^2+^ exists in the prepared material (Jahanshahi et al. 2020). The existence of Fe^3+^ is also supported by the presence of acidic protons present on the surface because they appear to compensate the extra negative charge generated in the lattice given that Ti is a tetravalent ion and Fe is a trivalent ion (Jahanshahi et al. 2020; Palanisamy et al. 2013).

Finally, in the Ti 2p spectrum (Fig. 4c), the peaks corresponding to binding energies of about 458 and 463.5 eV are due to Ti 2p_3/2_ and Ti 2p_1/2_, respectively, and then, they could be assigned to Ti^4+^ ions present in the TiO_2_ lattice (Güzelçimen et al. 2020). On the other hand, the small shoulder that appears at 456.5 eV corresponds a Ti^3+^ state, which is due to an oxygen deficiency in TiO_2_ (Iatsunskyi et al. 2021)_._ A second shoulder at higher binding energy (458.6 eV) arises from a Ti^4+^ state present in the Ti–O–Fe structure. At this point, it is important to note that the increase of core electron BE of Ti^4+^ and Fe^3+^ in the Ti–O–Fe bond may be due to the electron transfer from Ti^4+^ to Fe^3+^, caused by the Pauling electronegativity differential between Fe^3+^ (1.83) and Ti^4+^ (1.54) ions (Pham et al. 2014).

**Fig. 5:**
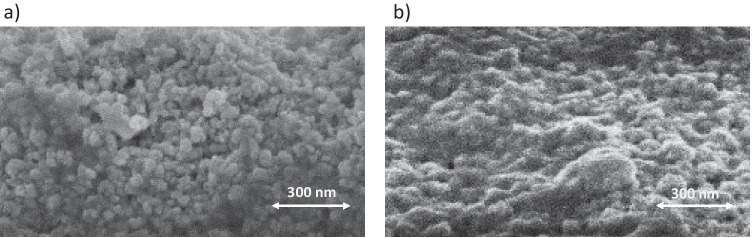
Scanning electron microscopic (SEM) micrograph of the TiO_2_ thin film (**a**) and 0.05%Fe-TiO_2_ thin film (**b**)

Once XPS demonstrates the incorporation of Fe to the TiO_2_ network, the effect of Fe^3+^ inclusion on the TiO_2_ films morphology was then analyzed. For this purpose, SEM micrographs of TiO_2_ and 0.05%Fe-TiO_2_ thin film are compared in Fig. 5a and b.

The SEM images show the effects of incorporating Fe^3+^ on the microstructure of TiO_2_. The micrograph of pure titania (Fig. 5a) indicates the existence of spherical particles of different sizes that are distributed homogenously through the matrix. The incorporation of Fe^3+^ seems to deform the degree of homogeneity of the system, and large number of particles with various shapes is detected. The micrograph of the Fe-TiO_2_ sample indicates the existence of small spherical particles similar to those observed for parent titania, in addition to various irregular particle agglomeration that results from the incorporation of Fe ions into titania structure. These results are in agreement and supported by other studies (Ahmed et al. 2013; Khasawneh et al. 2021).

Finally, the influence of the Fe^3+^ incorporation to the titania network on the optical band gap energy was evaluated. First, from the UV–Vis diffuse reflectance spectra the Kubelka–Munk function, F(R) was evaluated. Then, the Tauc method was applied to the function (F(R)∙E)^n^ versus E, the photon energy, with *n* = 1/2 for indirect semiconductors. This plot should exhibit a linear region where the relationship (F(R)⋅E)^1/2^ = k⋅(E − E_g_) applies. In this way, by extrapolating to zero this linear region, the band gap (*E*_*g*_) values corresponding to Fe-doped and undoped TiO_2_ NPs were obtained (Table 1). From the results presented in Table 1, we can infer that Fe^3+^ doping of titania semiconductor induces a reduction in its *E*_*g*_ value which determines the activity under visible light of the resulting material. In particular, it is observed that the effect becomes more pronounced as the Fe content is higher in the catalyst.

**Table 1:** Indirect band gap

Material	Indirect *E*_*g*_ (eV)
TiO_2_ 0.05% Fe-TiO_2_ 0.1% Fe-TiO_2_ 0.2% Fe-TiO_2_	3.19 ± 0.02 3.03 ± 0.03 2.95 ± 0.03 2.83 ± 0.03

Red shift is a frequent phenomenon observed in the transition metal doped II–IV semiconductor, and it could be ascribed to the production of oxygen vacancies generated, in this case, because of Fe^3+^ insertion in TiO_2_ lattice framework (Paul et al. 2021). For the investigated Fe-TiO_2_ materials and following the procedure described below, we propose an electronic band diagram where the band due to these oxygen vacancies is located in a position according with the results presented above. Thus, firstly, we used Eq. (3) and Eq. (4) to estimate the valence and conduction band positions (Jiang et al. 2017):

3 $${E}_{CB}=\chi -{E}^{c}-0.5{E}_{g}$$

4 $${E}_{g}={E}_{VB}-{E}_{CB}$$

where *E*^*C*^ = 4.5 eV is the scaling factor relating the normal hydrogen electrode scale (NHE) to absolute vacuum scale and $\chi$ is the absolute electronegativity of the material (5.81 eV and 5.85 eV for TiO_2_ and Fe-TiO_2_, respectively) that was determined knowing the values of electron affinity and ionization energies for Ti, O, and Fe elements (Habibi-Yangjeh and Shekofteh-Gohari 2017).

**Fig. 6:**
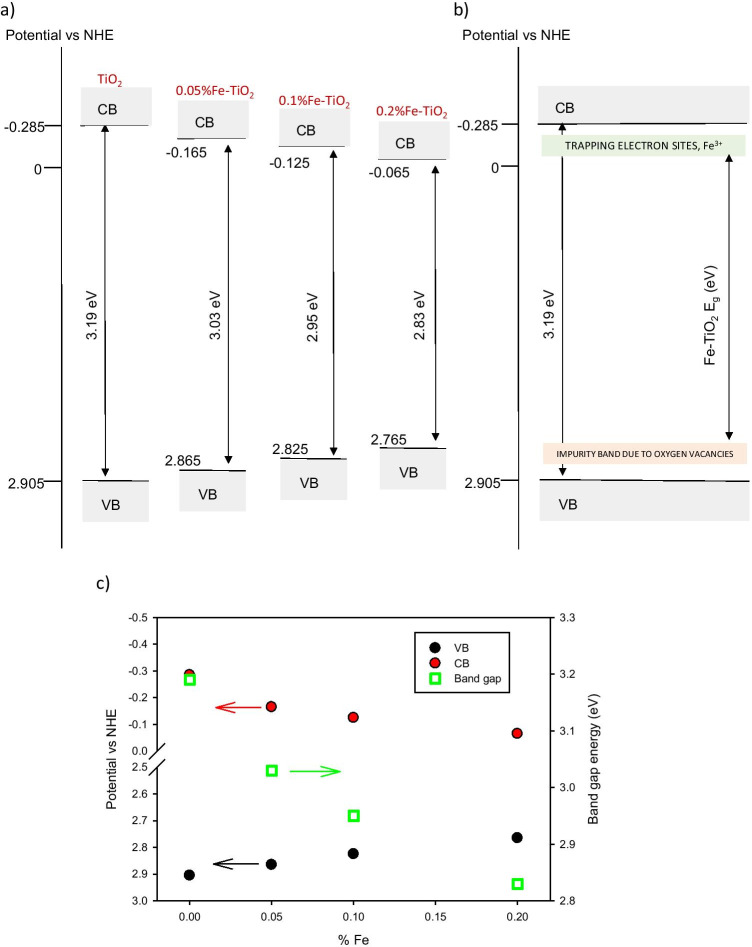
(**a**) Energy states diagrams. (**b**) A schematic presentation of band gap structure of pure and Fe-TiO_2_ samples. (**c**) Dependence of VB, CB positions and band gap energy with respect to Fe^3+^ content

As can be seen in Fig. 6a, the positions of VB and CB values are closer for those materials with higher Fe doping concentrations. It is also observed that both CB and VB positions move in the direction of less negative potential and less positive potential, respectively, when increasing the Fe content (Fig. 6c). This phenomenon confirms that Fe^3+^ ions have created an impurity band near the VB due to the oxygen vacancies, resulting in the effective reduction of the optical band gap (Paul et al. 2021) as is depicted in Fig. 6b.

Therefore, regarding the use of the studied materials in solar photocatalysis processes, by considering these band gap values, it could be expected that doping TiO_2_ NPs with a higher Fe content (0.20%) would lead to better results when solar photocatalysis processes will be applied. However, for such a statement, it is necessary to evaluate the efficiency of the proposed materials on the photocatalytic process.

### Photocatalytic studies

Sulfamethoxazole is a sulphonamide type synthetic antibiotic used for the elimination of bacteria causing different illnesses (Beltrán et al. 2008). SMT was selected in the present study as a model pollutant for photocatalytic degradation reactions because it is widely used and found in wastewater effluents and survives in wastewater as a hazardous compound (Beltrán et al. 2012). In this work, solar photochemical reactions for the degradation of SMT were used to compare the photocatalytic activity of the different Fe-TiO_2_ thin films. The objective of this analysis was to determine not only the optimal Fe^3+^ content, but also the most suitable thin film thickness to achieve the highest efficiency.

**Fig. 7:**
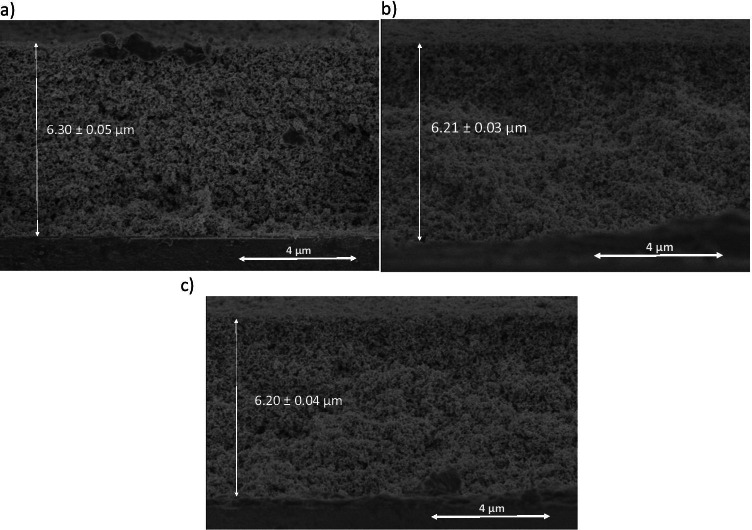
SEM images of cross section of the Fe-TiO_2_ thin film photocatalyst (**a**) 0.05%Fe-TiO_2_ thin film; (**b**) 0.1%Fe-TiO_2_ thin film; (**c**) 0.2%Fe-TiO_2_ thin film

To determine the optimal Fe^3+^ content, thin film photocatalysts with different doping level were prepared by depositing the same numbers of layers (in this case 15) regardless of the mole ratio Fe/TiO_2_, in order to reach a similar total thickness. SEM evaluation of the cross section presented in Fig. 7 confirms that the three samples with different Fe doses haves an analogous thickness around 6 µm.

**Fig. 8:**
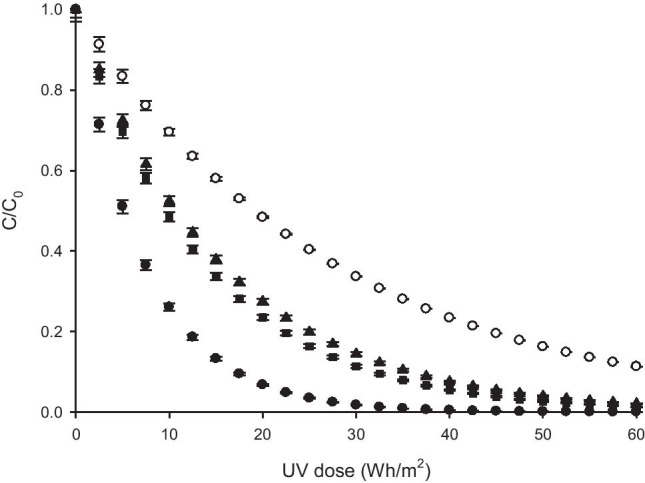
Degradation of SMT using Fe-TiO_2_ thin film photocatalysts with different %Fe^3+^ content. (black circle): 0.05%Fe-TiO_2_; (black square):0.1%Fe-TiO_2_; (black triangle): 0.2%Fe-TiO_2_; (white circle): TiO_2_

Solar photocatalysis processes were applied by using these thin films with similar thicknesses. Figure 8 represents the degradation behavior of SMT on solar irradiation in the presence of Fe-TiO_2_ thin film photocatalysts for the different %Fe content used. Results show the improvement in the photoactivity of Fe-TiO_2_ compared with bare TiO_2_ thin film with a thickness of 6.30 ± 0.04 µm. According to the literature, the presence of Fe^3+^ prevents the electron–hole recombination and promotes the generation of hydroxyl radicals (Khasawneh et al. 2021; Mesgari et al. 2012; Mohamed et al. 2019), and both effects contribute to improve the photoactivity of the doped material.

As mentioned before, by considering the lower band gap value obtained for the synthesized material with higher Fe doping content (0.2%wt.) (Table 1), it could be expected for this sample the higher efficiency in the solar photocatalysis process, given that solar radiation is more intense at the corresponding absorption wavelength. However, results showed in Fig. 8 reveal that by using the thin film with a lower Fe/TiO_2_ ratio, a higher SMT removal yield is achieved. The reason for this trend may be related to the fact that, for highest Fe doping levels, Fe mainly acts as recombination centers for photo-generated electrons and holes (Mesgari et al. 2012; Moradi et al. 2016; Nagaveni et al. 2004).

**Fig. 9:**
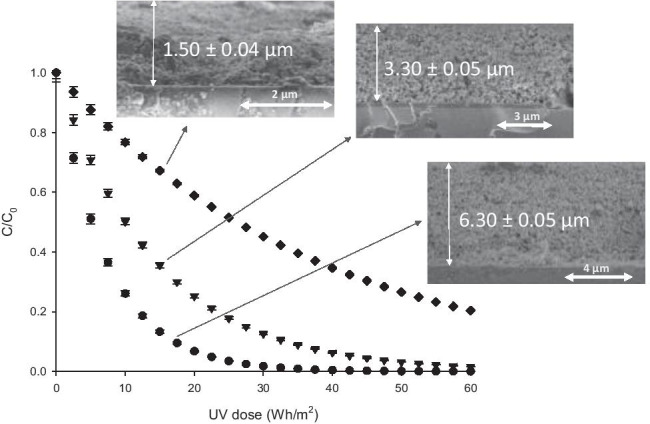
Degradation of SMT using 0.05%Fe-TiO_2_ thin film photocatalysts with different thickness. (black diamond): ~ 1.5 µm; (black inverted triangle): ~ 3.2 µm; (black circle): ~ 6.3 µm

Consequently, the 0.05%Fe-TiO_2_ sample was chosen in order to evaluate the influence of the film thickness on the efficiency for SMT elimination under solar irradiation. Specifically, thin films of different thicknesses were prepared by varying the number of successive layers deposited. The evolution of SMT concentration with the solar irradiation dose, shown in Fig. 9, indicates that, in the thickness range evaluated, the antibiotic removal performance is improved when increasing the film thickness.

Taking as a reference a degradation level of 75%, the solar dose required decreases from ~ 53 Wh/m^2^ for 1.5 μm to ~ 21 Wh/m^2^ when thickness is doubled and to ~ 10 Wh/m^2^ when is quadrupled. Then results in Fig. 9 indicate that solar photocatalysis efficiency increases with the film thickness. Additionally, experimental results show that the process follows first-order kinetics, according to Eq. (5), in which *t*_30W_ can be calculated from experimental data by using Eq. (2).

5 $$ln\frac{C}{{C}_{0}}=-k{t}_{30W}$$

**Fig. 10:**
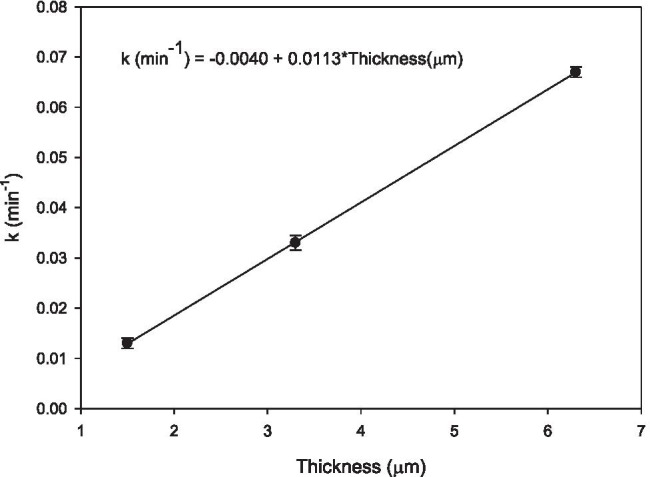
Relationship of the kinetic constant with the film thickness

First-order constants calculated for each sample are presented in Table 2. In this case, we find a linear relationship of the kinetic rate constant with the photocatalyst film thickness as evidenced in Fig. 10.

**Table 2:** Values of the apparent pseudo first order rate constant using 0.05% Fe-TiO_2_ thin films with different thickness in the oxidation of SMT in water

Thickness, µm	Layers deposited	k, min^−1^
1.50 ± 0.04 3.30 ± 0.05 6.30 ± 0.05	5 10 15	0.013 ± 0.001 0.033 ± 0.002 0.067 ± 0.001

**Fig. 11:**
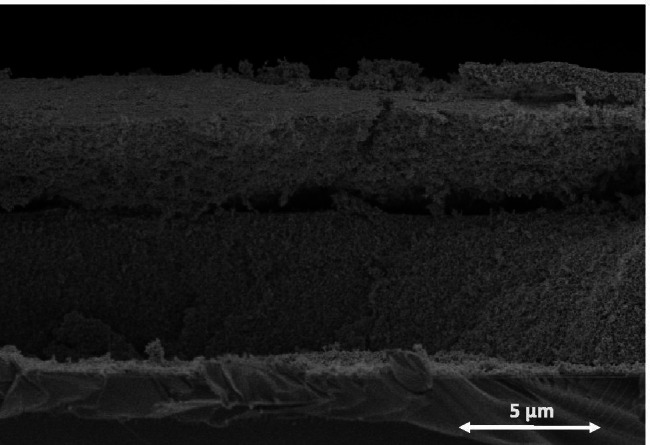
SEM images of cross section of Fe-TiO_2_ thin film photocatalyst with thickness around 8 µm

Thus, according to results obtained, in order to improve even more the photocatalytic performance, we tried to prepare thin film photocatalyts with a thickness as greater as possible. However, there is an upper limited for the achievable thickness that is superimposed by the integrity of the film as more layers are added. In fact, it is observed that when greater thicknesses were processed, adherence is not enough and material detachment occurred, as it is illustrated in SEM micrograph of Fig. 11.

On the other hand, since oxidation likely yields to first intermediates of similar hazardous character (Beltrán et al. 2008), removal of total organic carbon (TOC) can be more important than removal of the initial pharmaceutical compound. Thus, taking into account that the use of thin films with 0.05% Fe content and a thickness of approximately 6 µm allows to achieve the best SMT removal performance, changes of TOC with time were also followed by using this specific sample. It was found that, TOC follows a first-order kinetics which fits well for a pseudo first-order constant 3.7·10^−3^ ± 10^−4^ min^−1^.

Furthermore, it should be mentioned that, as a first approach of reusability of the prepared material, the best performing catalyst (0.05%Fe-TiO_2_) was checked by reusing in three cycles. No differences were detected in the photocatalytic efficiency of this sample; no differences were observed when the elimination of SMT was evaluated. Additionally, by applying the ferrozine method to the treated water, it was determined that iron total concentration in solution was equal to zero; that is, there was no iron leaching, confirming the stability of the prepared Fe-TiO_2_ thin films.

## Conclusions

High-resolution XPS demonstrates the existence of Fe^3+^ and suggests that no Fe^2+^ exists in the prepared material. The existence of Fe^3+^ is also supported by the presence of acidic protons present on the surface.

The incorporation of Fe^3+^ deforms the degree of homogeneity of the system, and large number of particles with various shapes is detected.

Fe^3+^ doping of titania semiconductor induces a reduction in its *E*_*g*_ value. The effect becomes more pronounced as the Fe content is higher in the catalyst. This phenomenon could be ascribed to the production of oxygen vacancies generated.

In the electronic band diagram of the prepared materials, the positions of VB and CB values are closer for those materials with higher Fe doping concentrations. Additionally, both CB and VB positions move in the direction of less negative potential and less positive potential, respectively, when Fe content is increased, confirming that the reduction of the optical band gap is due to the presence of oxygen vacancies.

Results show the improvement in the photoactivity of Fe-TiO_2_ compared with bare TiO_2_ thin film. The highest SMT removal yield is achieved by using the thin film with the lower Fe/TiO_2_ ratio.

Solar photocatalysis efficiency increases with the film thickness. However, there is an upper limited superimposed by the integrity of the film when layers are added.

Experimental results show that the elimination of SMT follows first-order kinetics, being linear the relationship of the kinetic constant with the film thickness.

A first approach to the reusability of the prepared material was checked by reusing the best performing catalyst (0.05%Fe-TiO_2_) in three cycles. Additionally, it was determined that there was not iron leaching, confirming the stability of the prepared Fe-TiO_2_ thin films.

## Data Availability

The datasets used and/or analyzed during the current study are available from the corresponding author on reasonable request.
